# Application of musculoskeletal ultrasound in quantitative assessment and rehabilitation of lower leg muscles in stroke patients

**DOI:** 10.3389/fneur.2026.1790982

**Published:** 2026-08-17

**Authors:** Chengrui Ma, Liansong Liu, Rongxing Yang, Ping Yao

**Affiliations:** Department of Rehabilitation, The Affiliated Hospital of Yunnan University/The Second People's Hospital of Yunnan Province, Kunming, Yunnan, China

**Keywords:** fiber length, muscle thickness, musculoskeletal ultrasound, pennation angle, rehabilitation outcome, stroke

## Abstract

**Objective:**

To investigate the application of musculoskeletal ultrasound in the quantitative assessment of lower leg muscles and its value in stratifying rehabilitation outcomes in patients with stroke.

**Methods:**

A single-center retrospective study was conducted, including 152 patients with stroke. Based on predefined composite functional criteria at 6 months after rehabilitation, patients were classified into favorable outcome (*n* = 105) and poor outcome (*n* = 47) groups. Baseline clinical characteristics (FMAS, Barthel Index, Brunnstrom stage, MMT, MAS) and MSUS parameters of the tibialis anterior (TA) and medial gastrocnemius (MG), including muscle thickness, fiber length, and pennation angle, were collected. Receiver operating characteristic (ROC) analysis, multivariable logistic regression, and hierarchical regression models were used to evaluate predictive performance.

**Results:**

Favorable outcomes were associated with greater TA and MG muscle architectural parameters (*P* < 0.05). ROC analysis showed moderate predictive ability for TA fiber length (AUC = 0.755) and MG fiber length (AUC = 0.652). The combined logistic model improved prediction (AUC = 0.801). Hierarchical analysis demonstrated that adding MSUS parameters to baseline functional scores increased predictive performance (AUC from 0.721 to 0.833).

**Conclusion:**

MSUS-derived muscle architecture, particularly fiber length, is associated with stroke rehabilitation outcomes and provides incremental predictive value beyond conventional functional assessments.

## 1 Introduction

Lower extremity motor dysfunction is a common complication following stroke. Manifestations such as muscle weakness, impaired motor control, and lower limb spasticity can lead to abnormal gait, balance disorders, and an increased risk of falls during ambulation (1). Traditional rehabilitation primarily focuses on motor relearning and constraint-induced movement therapy. While these approaches can improve lower limb function to some extent, the relatively standardized and procedural nature of the training often fails to account for individual patient variability, resulting in heterogeneous rehabilitation outcomes (2). Therefore, accurately predicting rehabilitation efficacy prior to initiating therapy is critically important, as it can provide valuable guidance for clinical management.

Musculoskeletal ultrasound (MSUS) is a noninvasive, low-cost, and repeatable technique that allows real-time visualization and quantitative assessment of muscle and periarticular soft tissues. In clinical practice, it has been widely used for the evaluation of musculoskeletal conditions and for guiding interventions such as botulinum toxin type A injection or specific acupuncture techniques in patients with post-stroke foot drop/inversion (3, 4). In addition to procedural guidance, MSUS can provide quantitative information on muscle architecture, including muscle thickness, fiber length, and pennation angle, which are closely related to muscle volume, extensibility, and force-generating capacity (5).

Among lower-limb muscles, the tibialis anterior (TA) and medial gastrocnemius (MG) are particularly important for ankle control, gait, and balance. The TA is the primary muscle responsible for ankle dorsiflexion and plays an essential role in foot clearance during walking, whereas the MG contributes substantially to ankle plantarflexion and postural stability. Previous studies have shown that architectural characteristics of the gastrocnemius are closely associated with lower-limb muscle performance and locomotor function (6). After stroke, neurological impairment, spasticity, and disuse may alter the architecture of these muscles, thereby affecting motor performance and rehabilitation potential (7). Accordingly, architectural characteristics of lower-limb muscles, particularly the gastrocnemius, may provide clinically meaningful information for functional assessment.

However, although MSUS has been increasingly used to assess muscle status and guide rehabilitation-related interventions, its value in predicting rehabilitation outcomes in patients with post-stroke lower limb motor dysfunction has been scarcely reported. Therefore, this study used MSUS to measure muscle thickness, fiber length, and pennation angle of the TA and MG before rehabilitation training, with the aim of exploring their value in quantitative assessment and rehabilitation outcome stratification in stroke patients.

## 2 Materials and methods

### 2.1 Clinical data

A total of 152 stroke patients diagnosed and treated at our hospital from March 2024 to February 2025 were enrolled in this retrospective study. Based on their rehabilitation efficacy at 6 months post-training, they were categorized into two groups: a favorable outcome group (*n* = 105) and a poor outcome group (*n* = 47). Rehabilitation efficacy was defined as fulfilling all the following criteria: a Manual Muscle Testing (MMT) grade of ≥3, a Modified Ashworth Scale (MAS) grade of 0–1, a Brunnstrom stage of ≥V, a Barthel Index score of >60, and a Fugl-Meyer Assessment Scale (FMAS) score of >95. All outcome assessments were performed at 6 months after completion of rehabilitation training. Patients not meeting these criteria were assigned to the poor outcome group.

Inclusion Criteria: (1) First-ever stroke confirmed by imaging (CT or MRI); (2) Age ≥ 18 years; (3) duration of post-stroke motor dysfunction ≥15 days; (4) presence of unilateral lower-limb motor dysfunction, manifested by muscle weakness, abnormal muscle tone, or impaired voluntary movement affecting standing, walking, or lower-limb functional training; and (5) stable vital signs.

*Exclusion Criteria*: (1) History of muscle relaxant use after stroke onset; (2) Presence of psychiatric disorders or significant cognitive impairment; (3) Diagnosis of deep vein thrombosis in the lower limbs; (4) Comorbid malignancy or severe cardiac, hepatic, or renal insufficiency.

A favorable outcome was defined as a composite indicator of clinically meaningful functional recovery after stroke, reflecting the simultaneous achievement of motor control, low spasticity, improved motor recovery stage, and functional independence. Patients were classified as having a favorable outcome only when all predefined thresholds (MMT ≥ 3, MAS ≤ 1, Brunnstrom stage ≥ V, Barthel Index > 60, and FMAS > 95) were met.

### 2.2 Data collection

For data collection, medical records of the study participants were reviewed. General information including sex, age, body mass index (BMI), duration of motor dysfunction, and stroke type was collected using Microsoft Excel spreadsheets. Pre-rehabilitation semi-quantitative motor function assessments included the Brunnstrom staging, MAS, MMT, Barthel Index, and FMAS. MSUS parameters, including muscle thickness, fascicle (muscle fiber) length, and pennation angle, were also recorded. The Brunnstrom staging (8) classifies motor recovery into six stages based on muscle strength and tone: stage I, flaccidity; stage II, spasticity; stage III, synergistic movement patterns; stage IV, partial dissociation of movements; stage V, dissociated movements; and stage VI, normal motor function. MAS (9) grades muscle tone as follows: grade 0, no increase in muscle tone; grade 1, slight increase in tone manifested by a catch and release or minimal resistance at the end of the range of motion (ROM); grade 1+, slight increase in tone manifested by a catch followed by minimal resistance through less than 50% of the ROM; grade 2, more marked increase in tone through most of the ROM, but affected parts easily moved; grade 3, considerable increase in tone, passive movement difficult; and grade 4, rigidity. MMT (10) is graded from 0 to 5: grade 0, no muscle contraction; grade 1, slight muscle contraction; grade 2, full ROM with gravity eliminated; grade 3, full ROM against gravity but without resistance; grade 4, full ROM against gravity with some resistance; and grade 5, full ROM against gravity with full resistance. The Barthel Index (11) assesses activities of daily living across 10 items, with total scores ranging from 0 to 100; a score >60 indicates basic independence in daily living. FMAS (12) evaluates motor function of the upper extremities (66 points) and lower extremities (34 points), with a total score of 100. Scores < 50 indicate severe motor dysfunction, 50–84 indicate marked motor dysfunction, 85–95 indicate moderate motor dysfunction, and ≥96 indicate mild motor dysfunction. In this retrospective study, the 6-month functional assessments were primarily used to classify patients into favorable and poor outcome groups according to predefined criteria, rather than being analyzed as continuous outcome variables. The MAS was assessed for the affected ankle plantar flexor muscle group during routine rehabilitation evaluation. In the present study, the MAS grade refers to the score of this specific muscle group rather than a composite or averaged score. This score was used both in baseline functional assessment and in outcome classification.

MSUS was performed within 1 week prior to rehabilitation training using a Konica Minolta portable ultrasound system equipped with a high-frequency linear-array transducer (5–17 MHz). Participants were examined in the supine position. For the tibialis anterior (TA), measurements were obtained at the proximal one-third of the line between the fibular head and the medial malleolus; for the medial gastrocnemius (MG), measurements were taken at the point of maximal muscle belly thickness. The transducer was positioned longitudinally along the muscle fibers with minimal pressure applied. All measurements were obtained with the muscles in a relaxed resting state. Muscle thickness, fascicle length, and pennation angle were each measured three times for both TA and MG, and the mean value was used for analysis. All examinations were performed by the same experienced sonographer with more than 5 years of musculoskeletal ultrasound experience using a standardized protocol. The time from stroke onset to baseline MSUS assessment was 34.3 ± 7.1 days. For statistical analysis, TA–MG parameters were calculated as follows: TA–MG thickness = (TA thickness + MG thickness)/2, TA–MG fascicle length = (TA fascicle length + MG fascicle length)/2, TA–MG pennation angle = (TA pennation angle + MG pennation angle)/2. Units were preserved during calculation (mm for thickness and fascicle length, and degrees for pennation angle). The TA and MG muscles were combined because both contribute to ankle control and gait function, and the averaged parameter was used to represent overall lower-leg muscle architecture.

### 2.3 Statistical analysis

Statistical analyses were performed using SPSS version 22.0 (IBM Corp., Armonk, NY, USA). The normality of continuous variables was assessed using the Shapiro–Wilk test prior to analysis. Continuous variables with approximate normal distribution are presented as mean ± standard deviation (SD) and were compared using the independent-samples *t* test, whereas categorical variables are presented as number (n) or percentage (%) and were compared using the chi-square (χ^2^) test. Effect sizes for continuous variables in group comparisons were calculated using Cohen's d. Pearson correlation analysis was used to assess associations between ultrasonographic parameters and continuous clinical variables with approximate normal distribution, including the Barthel Index and FMAS scores. Spearman correlation analysis was used for ordinal clinical variables, including MMT grades, MAS grades, and Brunnstrom stages. The strength of correlation coefficients was interpreted as weak for |r| < 0.30, moderate for 0.30 ≤ |r| < 0.50, and strong for |r| ≥ 0.50. A multivariable logistic regression model was constructed using the averaged TA–MG thickness, averaged TA–MG fiber length, and averaged TA–MG pennation angle to derive an integrated ultrasonographic parameter. Multicollinearity was assessed before model fitting, with a variance inflation factor (VIF) < 10 indicating no significant collinearity. Receiver operating characteristic (ROC) curve analysis was performed to evaluate the predictive performance of each ultrasonographic parameter and the integrated parameter for rehabilitation outcomes in stroke patients. Internal validation was conducted using the bootstrap method. Hierarchical logistic regression analysis was performed by first entering baseline functional scores (FMAS and Barthel Index), followed by musculoskeletal ultrasound parameters in a second step to evaluate incremental predictive value. A two-sided *P* value < 0.05 was considered statistically significant.

## 3 Results

### 3.1 Comparison of clinical characteristics

There were no significant differences between the favorable outcome and poor outcome groups in demographic and general clinical characteristics, including sex, age, BMI, duration of motor impairment, or stroke type (*P* > 0.05). However, significant between-group differences were observed in baseline motor function-related variables. Specifically, the proportion of patients with Brunnstrom stage II, MMT grade 1, and MAS grade 3 was significantly higher in the poor outcome group, whereas Barthel Index and FMAS scores were significantly lower than those in the favorable outcome group (*P* < 0.05; Table 1). These findings indicate that baseline functional status differed substantially between the two groups and was closely associated with subsequent rehabilitation outcomes.

**Table 1:** Comparison of baseline clinical and functional characteristics between the two groups.

Characteristics		Poor outcome group (*n* = 47)	Favorable outcome group (*n* = 105)	χ^2^*/t*	*P*	Cohen's d
Sex, [*n* (%)]	Male	26 (55.32)	55 (52.38)	0.113	0.737	_
	Female	21 (44.68)	50 (47.62)			
Age (years)		63.02 ± 4.96	63.02 ± 5.20	0.002	0.998	−0.000
BMI (kg/m^2^)		24.12 ± 2.79	24.09 ± 2.83	0.060	0.952	−0.011
Duration of motor impairment (d)		34.28 ± 7.04	34.33 ± 7.17	0.045	0.964	0.008
Stroke type [*n* (%)]	Hemorrhagic	18 (38.30)	35 (33.33)	0.352	0.553	–
	Ischemic	29 (61.70)	70 (66.67)			
Brunnstrom stage [*n* (%)]	Stage I	0	0	19.550	< 0.001	–
	Stage II	26 (55.32)	25 (23.81)			
	Stage III	15 (31.91)	32 (30.48)			
	Stage IV	6 (12.77)	48 (45.71)			
	Stage V	0	0			
	Stage VI	0	0			
MMT grade [*n* (%)]	Grade 0	0	0	15.147	< 0.001	–
	Grade 1	37 (78.72)	47 (44.76)			
	Grade 2	10 (21.28)	58 (55.24)			
	Grade 3	0	0			
	Grade 4	0	0			
	Grade 5	0	0			
MAS grade [*n* (%)]	Grade 0	0	0	17.761	< 0.001	–
	Grade 1	0	0			
	Grade 1^+^	6 (12.77)	43 (40.95)			
	Grade 2	17 (36.17)	40 (38.1)			
	Grade 3	24 (51.06)	22 (20.95)			
	Grade 4	0	0			
Barthel index (scores)		40.68± 6.76	46.91 ± 5.30	6.141	< 0.001	1.078
FMAS (scores)		62.83 ± 5.04	69.77 ± 6.23	6.717	< 0.001	1.179

BMI, body mass index; MMT, Manual Muscle Testing; MAS, Modified Ashworth Scale; FMAS, Fugl-Meyer Assessment Scale.Effect sizes for continuous variables are presented as Cohen's *d*; effect size was not calculated for categorical or ordinal variables.

### 3.2 Comparison of structural parameters of TA and MG muscles

**Figure 1:**
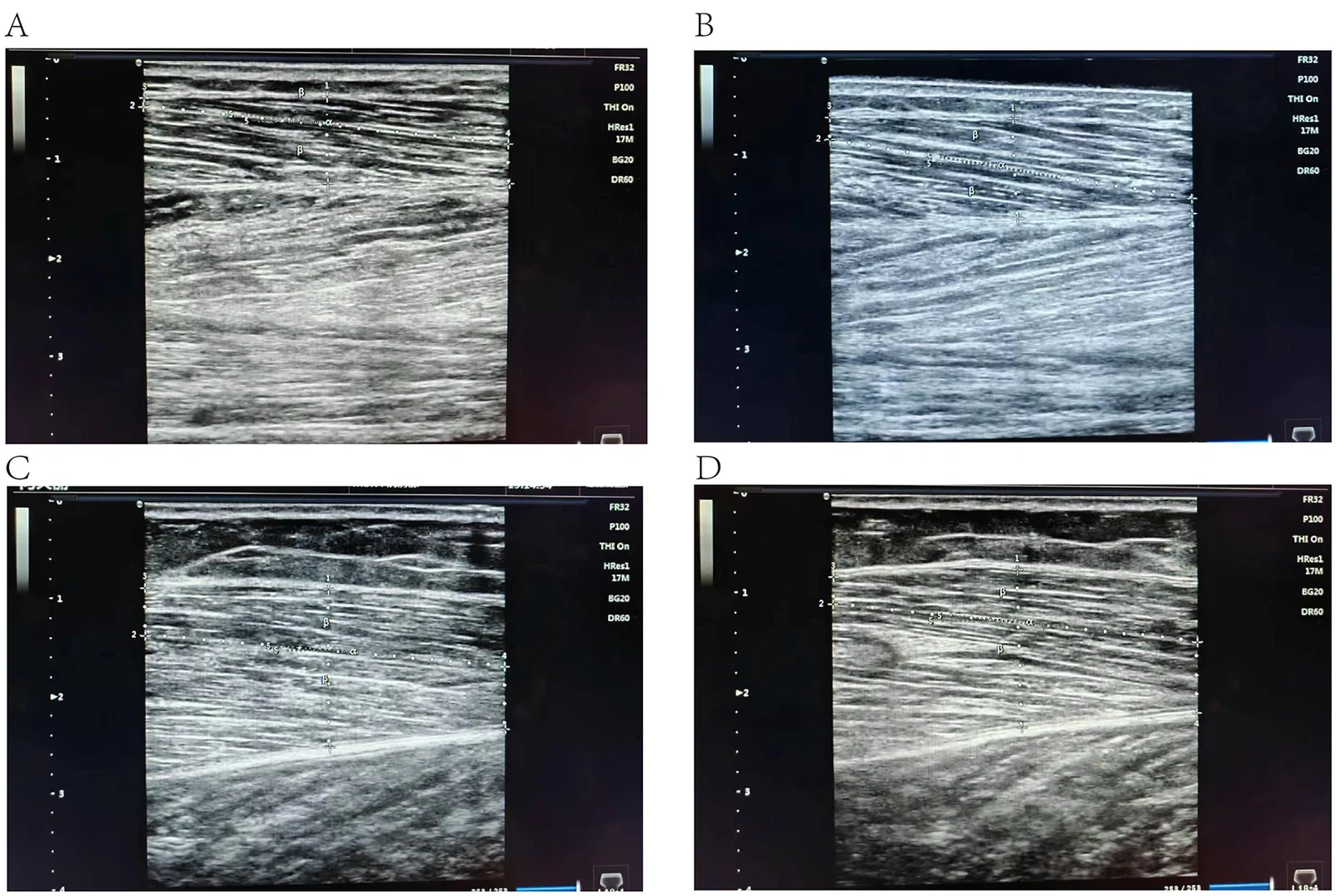
Representative musculoskeletal ultrasound images from a patient with stroke. **(A)** Tibialis anterior of the affected side at admission. **(B)** Tibialis anterior of the affected side after rehabilitation. **(C)** Medial gastrocnemius of the affected side at admission. **(D)** Medial gastrocnemius of the affected side after rehabilitation. The images illustrate the typical sonographic appearance of lower-leg muscle architecture and its changes following rehabilitation.

Muscle thickness, fiber length, and pennation angle for both the TA and MG were significantly lower in the poor outcome group compared to the favorable outcome group (*P* < 0.05; Table 2). The cut-off value of 15 mm refers to the averaged TA–MG thickness, rather than the individual TA or MG thickness shown separately in Table 2. Representative MSUS images from a patient are shown in Figure 1 to illustrate the sonographic appearance of the tibialis anterior and medial gastrocnemius on the affected side before and after rehabilitation.

**Table 2:** Comparison of structural parameters of the TA and MG muscles between the two groups.

Groups	TA			MG		
	Muscle thickness (mm)	Fiber length (mm)	Pennation angle (°)	Muscle thickness (mm)	Fiber length (mm)	Pennation angle (°)
Poor outcome group (*n* = 47)	8.55 ± 1.02	61.38± 3.74	6.11 ± 1.52	10.87 ± 1.26	32.26 ± 4.57	17.17 ± 1.90
Favorable outcome group (*n* = 105)	9.17 ± 1.37	65.75 ± 2.84	6.88 ± 1.36	12.26 ± 1.58	34.91 ± 5.12	18.24 ± 2.30
*t*	2.770	6.102	3.102	5.309	3.058	2.786
*P*	0.006	< 0.001	0.002	< 0.001	0.003	0.006
Cohen's d	0.486	1.071	0.544	0.932	0.537	0.489

TA, tibialis anterior; MG, medial gastrocnemius. Effect sizes for continuous variables are presented as Cohen's d. Averaged TA–MG values were calculated as the arithmetic mean of the corresponding measurements of the tibialis anterior and medial gastrocnemius on the affected side: averaged TA–MG parameter = (TA value + MG value)/2. Units were preserved (mm for thickness and fascicle length; ° for pennation angle).

### 3.3 Correlation analysis

Pearson correlation analysis revealed that the averaged TA–MG thickness, fiber length, and pennation angle were moderately to strongly positively correlated with both the Barthel Index and FMAS scores (all *P* < 0.001). Among these, correlations with the Barthel Index ranged from r = 0.569 to 0.640, and correlations with the FMAS score ranged from r = 0.507 to 0.660. Spearman correlation analysis showed that these averaged muscle parameters were moderately positively correlated with Brunnstrom stage (r = 0.345–0.461), weakly positively correlated with MMT grade (r = 0.194–0.222), and weakly negatively correlated with MAS grade (r = −0.296 to −0.283) (all *P* < 0.05) (Table 3).

**Table 3:** Correlation analysis between structural parameters of the TA and MG muscles and semi-quantitative motor function scales.

Variables	Averaged TA-MG thickness		Averaged TA-MG fiber length		Averaged TA-MG pennation angle	
	*r*	*P*	*r*	*P*	*r*	*P*
Barthel index	0.640	< 0.001	0.569	< 0.001	0.627	< 0.001
FMAS	0.606	< 0.001	0.660	< 0.001	0.507	< 0.001
Brunnstrom stage	0.461	< 0.001	0.367	< 0.001	0.345	< 0.001
MMT grade	0.222	0.006	0.194	0.017	0.222	0.006
MAS grade	−0.283	< 0.001	−0.296	< 0.001	−0.283	< 0.001

### 3.4 Construction of the integrated parameter model

A multivariable logistic regression model was used to construct the integrated ultrasonographic parameter. The averaged TA–MG thickness, averaged TA–MG fiber length, and averaged TA–MG pennation angle were entered simultaneously into the model based on their clinical relevance and associations with rehabilitation outcomes. Regression coefficients were estimated using maximum likelihood estimation. The final model was expressed as follows: Logit(P) = −14.952 + 0.150 × averaged TA–MG thickness + 0.144 × averaged TA–MG fiber length + 0.132 × averaged TA–MG pennation angle. The predicted probability of a favorable rehabilitation outcome was then calculated as:*P* = 1/[1 + *e*^∧^(−*Logit*(*P*))]. Multicollinearity diagnostics showed no evidence of problematic multicollinearity among the predictors, with variance inflation factor (VIF) values of 2.09 for averaged TA–MG thickness, 1.55 for averaged TA–MG fiber length, and 1.72 for averaged TA–MG pennation angle. Model fit was acceptable, with a Nagelkerke R^2^ of 0.255. In addition, the Hosmer–Lemeshow goodness-of-fit test showed no significant lack of fit (χ^2^ = 13.503, *P* = 0.096). The resulting predicted probability was defined as the integrated ultrasonographic parameter and was subsequently used for ROC analysis (Table 4).

**Table 4:** Multivariable logistic regression model for construction of the integrated ultrasonographic parameter.

Variables	B	SE	Wald	P	OR	95% CI		VIF
						Lower limit	Upper limit	
Averaged TA-MG thickness	0.150	0.160	0.878	0.349	1.162	0.849	1.592	2.09
Averaged TA-MG fiber length	0.144	0.048	9.040	0.003	1.155	1.051	1.268	1.55
Averaged TA-MG pennation angle	0.132	0.122	1.169	0.280	1.141	0.898	1.451	1.72
Constant	-14.952	3.473	18.531	< 0.001	–	–	–	–

### 3.5 Analysis of predictive efficacy

**Figure 2:**
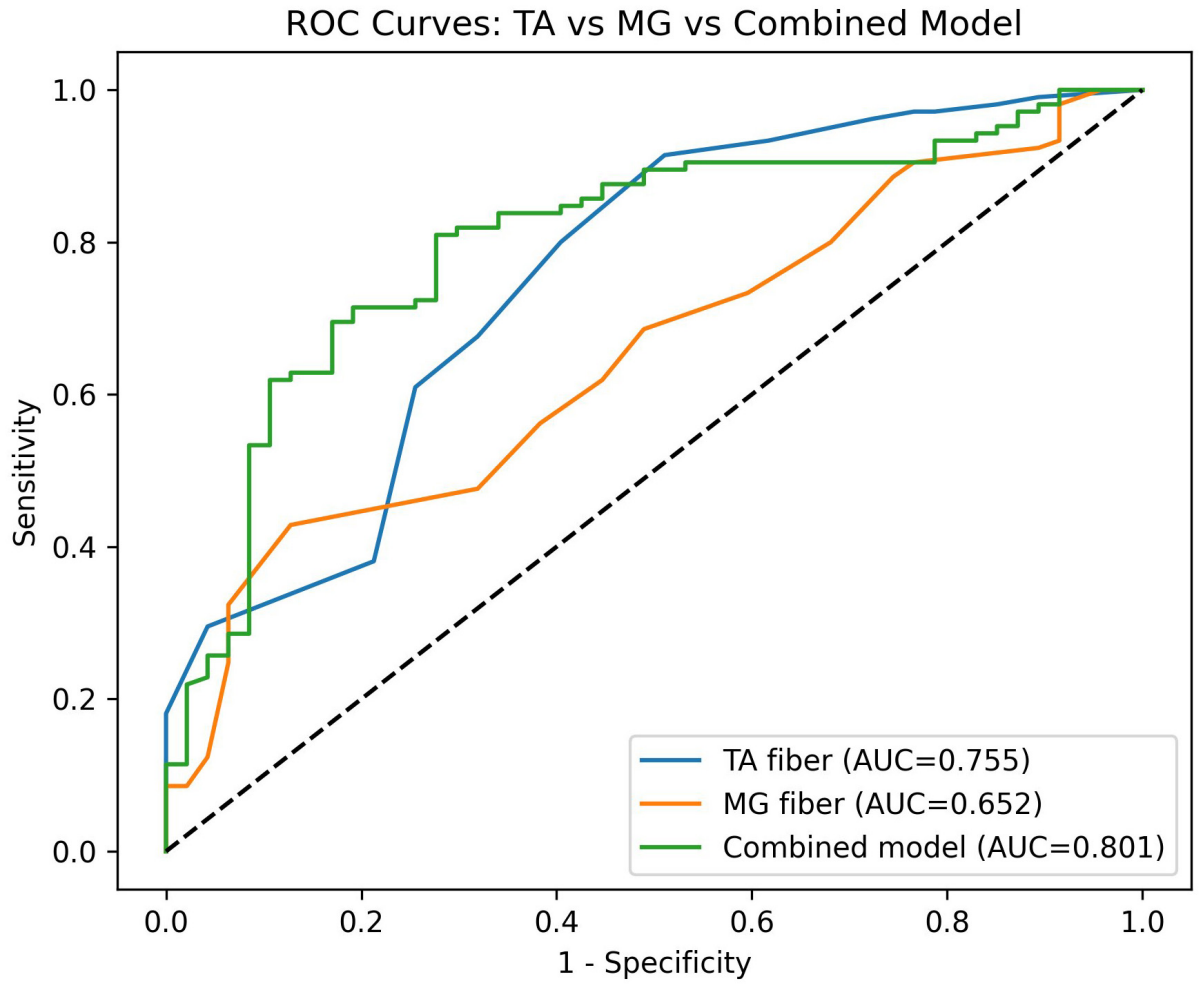
ROC curves for the predictive efficacy of TA fiber length, MG fiber length and combined model for rehabilitation outcome in stroke patients.

ROC curve analysis was performed to evaluate the predictive performance of tibialis anterior (TA) and medial gastrocnemius (MG) muscle architectural parameters, as well as a multivariable combined model, for rehabilitation outcomes in stroke patients. As shown in Figure 2 and Table 5, TA fiber length demonstrated moderate discriminative ability (AUC = 0.755), while MG fiber length showed relatively lower predictive performance (AUC = 0.652). Additional musculoskeletal ultrasound parameters (muscle thickness and pennation angle) were included in Table 5 for completeness, while Figure 2 displays only representative ROC curves for fiber length and the integrated model to improve clarity. Among individual parameters, MG muscle thickness exhibited relatively higher discriminative ability (AUC = 0.766), suggesting that structural adaptation of the gastrocnemius may be more sensitive to rehabilitation-related changes than fiber orientation parameters. Both TA and MG pennation angles showed limited predictive value (AUC = 0.649 and 0.633, respectively), indicating that angular architecture alone may be less strongly associated with functional outcomes compared with muscle thickness and fiber length. Importantly, the multivariable logistic regression–derived combined model incorporating TA and MG muscle thickness, fiber length, and pennation angle demonstrated the best predictive performance, with an AUC of 0.801. The optimal cutoff value of the model was 0.49, with a sensitivity of 87.6% and specificity of 76.6%, indicating good discriminative ability. Overall, ROC analysis demonstrated a stepwise improvement in predictive performance from single-muscle parameters to the integrated model, highlighting the advantage of combining TA and MG muscle architecture for outcome stratification in stroke patients (Figure 2, Table 5).

**Table 5:** Predictive performance of MSUS parameters.

Parameter	AUC	95% CI	Cut-off	Sensitivity (%)	Specificity (%)	Youdenindex
TA fiber length	0.755	0.671–0.839	62.8 mm	72.3	69.5	0.418
MG fiber length	0.652	0.553–0.751	33.9 mm	65.9	61.7	0.276
TA thickness	0.655	0.553–0.757	8.9 mm	63.8	64.3	0.281
MG thickness	0.766	0.684–0.848	11.9 mm	71.4	73.2	0.447
TA pennation angle	0.649	0.545–0.753	6.3°	68.1	60.4	0.284
MG pennation angle	0.633	0.528–0.738	17.8°	66	58.7	0.247
Combined model	0.801	0.771–0.895	0.49	87.6	76.6	0.642

### 3.6 Hierarchical logistic regression analysis

To evaluate the incremental predictive value of musculoskeletal ultrasound (MSUS) parameters beyond baseline clinical functional status, a hierarchical logistic regression model was constructed.

In Model 1, baseline functional scores including the Fugl-Meyer Assessment Scale (FMAS) and Barthel Index were entered. The model showed moderate predictive ability for rehabilitation outcomes (AUC = 0.721). In Model 2, MSUS-derived parameters (TA fiber length, MG fiber length, muscle thickness, and pennation angle) were added to the baseline model. After inclusion of MSUS parameters, model performance significantly improved (AUC increased from 0.721 to 0.833), and Nagelkerke R^2^ increased accordingly, indicating improved explanatory power. These findings suggest that MSUS parameters provide additional prognostic information beyond conventional functional assessment scales.

## 4 Discussion

Stroke is a common cerebrovascular disease in clinical practice, and motor dysfunction remains one of its most frequent sequelae. Clinical surveys indicate that more than two-thirds of patients with stroke develop post-stroke sequelae (13), among which motor dysfunction is the most prevalent. Previous studies have reported that lower limb motor dysfunction occurs in approximately 70.00%−80.00% of stroke survivors (14). In clinical practice, patients with post-stroke lower limb dysfunction are commonly treated with conventional rehabilitation therapies, including active and passive lower limb exercises, sitting and standing balance training, weight-shifting exercises, and gait training. Although these interventions can improve lower limb motor function, some patients remain unable to walk independently after completing rehabilitation, which may be attributable to the lack of individualized and targeted training in conventional rehabilitation programs (15). Specifically, insufficient training intensity in some patients may fail to adequately stimulate the neuromuscular system, thereby limiting rehabilitation efficacy, whereas excessive training intensity or frequency in others may lead to adverse effects such as fatigue and muscle strain, ultimately hindering the rehabilitation process (16, 17). Under these circumstances, identifying objective indicators that reflect lower limb muscle status before rehabilitation is of practical importance for individualized rehabilitation planning.

In the multivariable logistic regression model, only muscle fiber length demonstrated independent statistical significance, whereas muscle thickness and pennation angle did not reach statistical significance (*P* > 0.05). This suggests that the predictive performance of the integrated model was primarily driven by fiber length, which may reflect its stronger association with muscle contractile capacity and functional recovery potential after stroke. Nevertheless, although thickness and pennation angle were not independently significant, their inclusion contributed to the overall model stability and improved discriminatory performance, indicating a complementary rather than isolated predictive role. Although muscle thickness and pennation angle were not independently significant, their inclusion improved overall model discrimination, supporting their auxiliary role in muscle architecture–based prediction.

The TA is the primary muscle responsible for ankle dorsiflexion, whereas the MG plays an important role in ankle plantarflexion, postural control, and gait propulsion (18). After stroke, impaired descending motor drive, altered muscle activation, spasticity, and disuse may lead to substantial architectural remodeling of these lower-limb muscles. In the present study, reduced muscle thickness, fascicle length, and pennation angle in the poor outcome group may reflect muscle atrophy, impaired contractile capacity, and maladaptive remodeling after stroke. These findings are consistent with previous ultrasound-based studies showing that paretic lower-limb muscles exhibit altered muscle architecture compared with the unaffected side or with healthy individuals, and that such changes are associated with poorer clinical motor performance (19). Liu et al. also reported that TA and MG architectural parameters changed after body weight-supported treadmill training in patients with subacute stroke, indicating that these ultrasound-derived features may reflect rehabilitation-related neuromuscular recovery (20). Similarly, Wang et al. found that post-stroke individuals had substantial alterations in ankle plantarflexor and dorsiflexor architecture, further supporting the functional relevance of these parameters (7). Together, these findings support the view that ultrasound-assessed muscle architecture is not merely descriptive, but may also provide clinically meaningful information for rehabilitation evaluation.

Another important finding of this study is that the integrated ultrasonographic parameter combining muscle thickness, fascicle length, and pennation angle showed better discriminative performance than any single parameter alone. Although few previous studies have investigated exactly the same integrated model, this result is biologically plausible because these three parameters reflect different but complementary aspects of muscle architecture. This interpretation is also supported by previous studies showing that ultrasound-derived assessment of TA and gastrocnemius architecture can provide objective and clinically useful information in stroke rehabilitation and functional evaluation (21).

Our findings should also be interpreted in the context of the broader literature on prediction of lower-limb recovery after stroke. Previous studies, such as the TWIST tool developed by Stinear and colleagues, have shown that early bedside clinical measures can be used to predict the probability and timing of independent walking after stroke (22). In parallel, imaging-based approaches, including MRI biomarkers of the brain and lower-limb muscles, have also been explored for predicting lower-limb motor recovery (23). Compared with these approaches, the present MSUS-based method may offer several practical advantages. MSUS is simple, low-cost, portable, repeatable, and can be performed at the bedside without radiation exposure or the resource demands associated with MRI. In addition, unlike general clinical prediction tools, MSUS provides direct quantitative information on peripheral muscle architecture, including muscle thickness, fascicle length, and pennation angle, which may be particularly relevant for distal lower-limb function, ankle control, and gait. Therefore, the present approach may serve as a practical adjunct to existing clinical and imaging-based prediction strategies (24).

MSUS itself has several practical advantages, including noninvasiveness, low cost, bedside accessibility, and repeatability (25). In addition, previous studies have demonstrated acceptable reliability and clinical applicability of ultrasound-based musculoskeletal assessment, supporting its feasibility in rehabilitation evaluation (26).

Nevertheless, this study has several limitations that should be acknowledged. First, this was a single-center retrospective study, which may limit the generalizability of the findings. Second, although 6-month functional criteria were used to define rehabilitation outcomes, detailed itemized post-rehabilitation scores and change-from-baseline data were not available for all patients, limiting quantitative assessment of the magnitude of functional recovery. Third, because baseline functional differences existed between groups, including Barthel Index and FMAS, the present study could not fully exclude residual confounding, and the incremental predictive value of musculoskeletal ultrasound parameters should be interpreted with caution. Fourth, established neurological severity measures such as the National Institutes of Health Stroke Scale (NIHSS) and modified Rankin Scale (mRS) were not available, which limits adjustment for baseline stroke severity. Finally, formal intra- and inter-rater reliability analyses of musculoskeletal ultrasound measurements were not performed, which may affect the reproducibility assessment. Future prospective multicenter studies with larger sample sizes and standardized imaging protocols are needed to validate these findings.

In conclusion, MSUS assessment of muscle thickness, fiber length, and pennation angle may have potential value in evaluating lower-limb muscle status and discriminating rehabilitation outcomes in patients with stroke, and may provide useful support for individualized rehabilitation planning.

## Data Availability

The original contributions presented in the study are included in the article/supplementary material, further inquiries can be directed to the corresponding author.
